# Characterization of Bovine Intraepithelial T Lymphocytes in the Gut

**DOI:** 10.3390/pathogens12091173

**Published:** 2023-09-19

**Authors:** Akanksha Hada, Lei Li, Anmol Kandel, Younggeon Jin, Zhengguo Xiao

**Affiliations:** Department of Animal and Avian Sciences, University of Maryland, College Park, MD 20742, USA; hada@umd.edu (A.H.); lixxx242@umd.edu (L.L.); akandel1@umd.edu (A.K.); ygjin@umd.edu (Y.J.)

**Keywords:** cattle, intraepithelial T lymphocytes, T-IELs, TCRαβ, TCRγδ, CD4, CD8, IFNγ, IL17A, TGFβ1

## Abstract

Intraepithelial T lymphocytes (T-IELs), which constitute over 50% of the total T lymphocytes in the animal, patrol the mucosal epithelial lining to defend against pathogen invasion while maintaining gut homeostasis. In addition to expressing T cell markers such as CD4 and CD8, T-IELs display T cell receptors (TCR), including either TCRαβ or TCRγδ. Both humans and mice share similar T-IEL subsets: TCRγδ^+^, TCRαβ^+^CD8αα^+^, TCRαβ^+^CD4^+^, and TCRαβ^+^CD8αβ^+^. Among these subsets, human T-IELs are predominantly TCRαβ^+^ (over 80%), whereas those in mice are mostly TCRγδ^+^ (~60%). Of note, the majority of the TCRγδ^+^ subset expresses CD8αα in both species. Although T-IELs have been extensively studied in humans and mice, their profiles in cattle have not been well examined. Our study is the first to characterize bovine T-IELs using flow cytometry, where we identified several distinct features. The percentage of TCRγδ^+^ was comparable to that of TCRαβ^+^ T-IELs (both ~50% of CD3^+^), and the majority of bovine TCRγδ^+^ T-IELs did not express CD8 (CD8^−^) (above 60%). Furthermore, about 20% of TCRαβ^+^ T-IELs were CD4^+^CD8αβ^+^, and the remaining TCRαβ^+^ T-IELs were evenly distributed between CD4^+^ and CD8αβ^+^ (~40% of TCRαβ^+^ T-IELs each) with no TCRαβ^+^CD8αα^+^ identified. Despite these unique properties, bovine T-IELs, similar to those in humans and mice, expressed a high level of CD69, an activation and tissue-retention marker, and a low level of CD62L, a lymphoid adhesion marker. Moreover, bovine T-IELs produced low levels of inflammatory cytokines such as IFNγ and IL17A, and secreted small amounts of the immune regulatory cytokine TGFβ1. Hence, bovine T-IELs’ composition largely differs from that of human and mouse, with the dominance of the CD8^−^ population among TCRγδ^+^ T-IELs, the substantial presence of TCRαβ^+^CD4^+^CD8αβ^+^ cells, and the absence of TCRαβ^+^CD8αα^+^ T-IELs. These results provide the groundwork for conducting future studies to examine how bovine T-IELs respond to intestinal pathogens and maintain the integrity of the gut epithelial barrier in animals.

## 1. Introduction

Gastrointestinal (GI) disorders, ranging from acute infections to chronic inflammatory diseases, present considerable economic and health implications in humans as well as in cattle. Intraepithelial T lymphocytes (T-IELs), which make up about 90% of all intestinal intraepithelial lymphocytes and 50–60% of total T lymphocytes in humans, play a critical role in pathogen clearance and gut homeostasis, and could be a central target for developing intervention strategies against GI diseases [1,2,3,4,5,6,7]. However, our understanding of the composition and function of bovine T-IELs is limited by technological constraints and the delayed availability of specific research tools [8,9,10,11,12,13,14,15,16,17,18,19,20,21].

In contrast to the peripheral T cells, T-IELs are found in the antigen-abundant mucosal environment and possess unique homing molecules, activation states, and effector functions [3,4,5,22,23,24,25,26,27,28,29,30,31,32,33]. For instance, T-IELs express high levels of gut-homing molecules such as CD103 and CCR9, while expressing very low levels of lymphoid trafficking molecules like CD62L [1,23,34,35,36,37,38,39,40,41,42]. Furthermore, T-IELs are routinely exposed to both commensals and pathogens, and their behaviors are affected by a variety of distinct factors, including dietary nutrients such as glucose and vitamins, microbial metabolites like indole derivatives, and neighboring cells such as intestinal epithelial cells (IECs) [3,6,22,24,25,26,27,28,29,30,31,43,44,45,46,47,48,49,50,51,52,53,54,55,56,57,58,59,60,61,62,63,64,65,66,67,68,69]. Unlike peripheral T cells, which are mostly naive and are activated upon sensing antigens presented by antigen-presenting cells in the context of an MHC molecule, T-IELs possess a highly restricted T cell receptor (TCR) repertoire and typically display a semi-activated phenotype [2,32,33,70,71,72,73,74,75,76,77,78]. Understanding the unique features and compositions of T-IELs is essential to deepen our knowledge of mucosal immune responses within the gut of cattle and to advance the development of targeted immune interventions.

T-IELs are categorized into two groups based on their origin: natural and induced [79]. Natural T-IELs comprise the TCRαβ^+^CD8αα^+^ and TCRγδ^+^ subsets, both of which exhibit innate-like features [22,42,61,80,81,82,83,84,85,86]. After encountering endogenous self-antigens through their TCR in the thymus or extrathymic organs, these natural T-IELs rapidly populate the T-IEL compartment [22,80,87,88,89,90]. Despite the fact that the potential extrathymic origin of the TCRαβ^+^CD8αα^+^ subset remains a topic of debate, it has been generally accepted that they originate in the thymus [22,42,80,87,91]. Both natural T-IEL subsets are considered to be pathogen non-specific due to their selection from self-antigen recognition [22,92,93,94,95]. Meanwhile, the TCRαβ^+^CD8αα^+^ subset expresses natural killer (NK) receptors such as NKG2A/CD94 and the Ly49 family, which modulate immune responses by detecting altered MHC class I molecules on compromised cells and by producing immune regulatory cytokines like IL-10 and TGFβ1 [79,96,97]. On the other hand, TCRγδ^+^ cells depend on IEC-intrinsic MyD88 signaling, which induces TCRγδ^+^ T-IELs to produce antibacterial lectin RegIIIγ along with cytokines like IFNγ and IL-13, facilitating the clearance of pathogens such as *Salmonella*, *Toxoplasma*, *Listeria*, and *Nippostrongylus* [2,3,4,5,6,24,57,79,98,99,100,101,102,103,104,105,106,107]. In contrast to their natural counterparts, induced T-IELs comprise the TCRαβ^+^CD4^+^ and TCRαβ^+^CD8αβ^+^ subsets, which originate from antigen-stimulated peripheral T cells [22,79] and function predominantly in a pathogen-specific manner. For instance, in both humans and mice, TCRαβ^+^CD4^+^ T-IELs contribute to anti-inflammatory responses by producing immune regulatory cytokines, such as IL-10 [63,108,109], and the TCRαβ^+^CD8αβ^+^ subset initiates cytotoxic effector memory responses against pathogens such as simian immunodeficiency virus, lymphocytic choriomeningitis virus, *Toxoplasma*, and *Giardia* through TCR [2,98,110,111,112,113,114]. Interestingly, human TCRαβ^+^CD8αβ^+^ T-IELs can also respond in an antigen non-specific manner by expressing NK receptors in conditions like celiac disease whereas, in mice, it is the TCRαβ^+^CD8αα^+^ subset that performs this function [1,22,40,61,79,98,115]. Despite the overall functional similarities between the subtypes, mice predominantly have natural T-IELs, while induced T-IELs are more common in humans [1,2,79].

As ruminants, cattle possess a four-chambered stomach and a significantly longer intestine, differing from humans and mice not only in terms of gut environment but also in variations in the immune system [79,116,117,118,119,120,121]. For instance, the TCRγδ^+^ population constitutes approximately 60 percent of the total peripheral blood mononuclear cells (PBMC) in young cattle, a proportion significantly higher compared to the 2–15% found in humans and mice [120,122,123,124]. Moreover, during pathogen infections, bovine CD4^+^ T cells in blood and lymph nodes mount immune responses that differ partially from those of humans and mice [117,118]. Despite these anatomical and immunological differences, bovine T-IELs have been reported to respond to various infections caused by viruses, bacteria, and nematodes in a manner similar to humans and mice, suggesting crucial roles in immune responses, immune tolerance, and epithelial healing [8,10,11,13,14,16,21,125,126]. For example, T-IELs in cattle, including CD4^+^ and CD8^+^ cells, proliferate in response to various pathogens such as *Cryptosporidium parvum* and *Escherichia coli* [8,9,10,19,127]. Conversely, a decrease in the CD4^+^ and CD8^+^ T-IEL population correlates with increased disease severity [12]. Historically, studies on T-IELs in cattle have been limited by resources and technology. Recent advancements and improved research tools provide a fresh opportunity to deepen our understanding of bovine T-IELs [10,11,15,16,17,18,19,20,21]. We investigated the T-IELs in the bovine small intestine, particularly in the jejunum, using flow cytometry. Our findings highlight that while bovine T-IELs do exhibit some similarities with those in humans and mice, they also display unique composition patterns. The uniqueness is especially apparent in the even distribution of the TCRγδ^+^ and TCRαβ^+^ T-IEL subsets, the presence of distinct subsets such as TCRαβ^+^CD4^+^CD8αβ^+^ and TCRγδ^+^CD8^−^, and the absence of TCRαβ^+^CD8αα^+^ cells. Despite these variations, bovine T-IELs, similar to their human and mouse counterparts, continue to express T-IEL markers and produce various cytokines in the gut.

## 2. Materials and Methods

### 2.1. Cattle

Since 1958, the Wye Research and Education Center, University of Maryland Experimental Station (Queenstown, MD, USA) [32,128], has maintained the Wye Angus as a closed herd. All cattle were born between January and April, weaned at approximately six months of age, and had access to pastures before weaning. After weaning, the calves were randomly allocated to receive either grain or grass. The grain-fed group had no access to pasture and was fed a diet consisting of maize silage, corn cobs, and soybeans with added trace elements. The grass-fed group was allowed to graze on alfalfa-dominated pasture during the grazing season and hay during the winter [129]. Li et al. (2019) found that grain-fed steers attained market weight at approximately 14 months of age [130]. The animals in this investigation were grain-fed and euthanized in a commercial facility (George G Ruppersberger & Sons, Baltimore, MD, USA). Blood, lymph nodes, and entrails were collected at the slaughterhouse. Animal Care and Use Protocols were authorized by the UMD Institutional Animal Care and Use Committee (R-FEB-18-06 and R-JAN-21-02). All procedures were carried out in accordance with the relevant guidelines and regulations.

### 2.2. Isolation of Cells from Lymph Nodes and Blood

The inguinal lymph nodes (IGLN) were cut into 2–3 mm^3^ fragments and subjected to mechanical disruption and digestion in 5 mL of RP10 medium supplemented with 400 U/mL of V Collagenase, 0.1 mg/mL of DNase, and 2.5 U/mL of hyaluronidase at a temperature of 37 °C for a duration of 2 h [15,21]. The single-cell suspension obtained was subjected to cell counting and antibody staining. Peripheral blood mononuclear cells (PBMCs) were similarly performed as in our previous reports [130,131,132]. Blood was collected from the jugular vein using EDTA-coated vacutainers (Becton Dickinson Vacutainer Systems, Franklin Lakes, NJ, USA) and transferred to 15 mL conical containers (Fisher Scientific, Pittsburgh, PA, USA), which were centrifuged at 1200× *g*-force (G) for 30 min. Following centrifugation, the buffy coat at the interface was carefully collected into a new 15 mL tube and re-suspended in 8 mL of 1× phosphate-buffered saline (PBS) (Fisher Scientific, Fair Lawn, NJ, USA). Then, 5 mL of lymphocyte separation medium (LSM) with a density of 1.077 g/mL (Corning, Manassas, VA, USA) was added, followed by 30 min of centrifugation at 900G with break off. The interface’s second buffy coat was collected and cleaned twice with PBS. The cell pellet was resuspended in 5 mL Allos medium following the final rinsing, and a small aliquot was used for cell counting. Allos media was RPMI-1640 supplemented with FCS (10%), HEPES (10 mM), MEM non-essential amino acid (1×), sodium pyruvate (1 mM), penicillin and streptomycin (100 U/mL), L-glutamine (2 mM), and 2-mercaptoethanol (50 μM) (all from Mediatech, Manassas, VA, USA).

### 2.3. T-IEL Isolation

The T-IEL isolation protocol for the abomasum was adapted from [21], and for the jejunum and ileum, it was based on [15]’s method for mucosal small intestine T-IEL isolation. Both procedures included minor modifications. Briefly, approximately 100 g of the abomasum sample and about 10 cm sections each of the jejunum and ileum were gently washed, dipped in 95% ethanol, and then rinsed in CMF Hanks (Corning, NY, USA) containing 2 mM DTT (Fisher Bioreagents, Ottawa, ON, Canada) to remove surface mucus. These sections were subsequently cut into 1 cm^2^ pieces. These tissue pieces were then incubated in 50 mL of CMF Hanks with 2% FBS at 200 rpm at 37 °C for 30 min (SI-600, LAB Companion, Daejeon, Republic of Korea). After each incubation, the supernatant was collected, and the procedure was repeated twice using fresh CMF Hanks with 2% FBS. The accumulated supernatants were pooled together into a 200 mL beaker, allowing the epithelial cells to settle for 10 min. Without disturbing the sediment, the supernatants were decanted into new 50 mL conical tubes (Cellstar, Greiner Bio-one, NC, USA) and then strained using 70 µm cell strainers (VWR, Radnor, PA, USA). The tubes were then centrifuged at 500 G for 15 min at 22 °C. The resulting pellets were resuspended in 40% isotonic Percoll (Cytiva, Upsala, Sweden) in RPMI supplemented with 5% FBS. These suspensions were carefully layered over 80% isotonic Percoll and centrifuged at 600 G for 30 min at 22 °C without break. Cells from the 40%/80% interface were collected into 15 mL conical tubes. They were then washed with RPMI1640 containing 5% FBS, followed by centrifugation. The final cell pellet was resuspended for counting and subsequent experiments.

### 2.4. Antibodies and Reagents

All the antibodies used in this study are listed in the following tables: Supplementary Table S1 (primary antibodies) and Supplementary Table S2 (secondary antibodies and isotype controls). Staining buffer (SB) was 1 × PBS with 2% FBS, and fix solution was 4% paraformaldehyde (W/V) in 1 × PBS with pH 7.4. Intracellular staining permeabilization wash buffer (P/W) (BioLegend, San Diego, CA, USA) was purchased and used following the manufacturer’s instruction.

### 2.5. FACS

Approximately 10^6^ cells were allocated to FACS tubes (Fisher Scientific, Falcon, USA) for surface staining. These cells were sequentially stained with primary antibodies (Supplementary Table S1), followed by secondary and fluorescence-conjugated antibodies (Supplementary Table S2). Each staining step involved a 25 min incubation at 4 °C and was followed by a wash with SB to remove any unbound antibodies. After the final wash, cells were incubated with a fix solution for 15 min at 4 °C. This was followed by another SB wash, and then the cells were resuspended in 100 μL SB for analysis with the FACSCalibur™ flow cytometer.

For the intracellular staining, 10^6^ cells per sample were aliquoted and resuspended in 1 mL of complete Allos medium supplied with a cell activation cocktail (Bio-techne, Minneapolis, MN, USA) to achieve a final concentration of 1× in the medium to activate T cells while ensuring retention of the cytokines they produce within the cells [133,134]. This cocktail consisted of monensin sodium salt (1.5 mM), Phorbol 12-myristate 13-acetate (0.0405 mM), and Ionomycin calcium salt (0.67 mM). Additionally, Brefeldin A (BFA) (BioLegend, San Diego, CA, USA) alone was used as a control to determine the baseline production of cytokines in the absence of an added stimulus [135,136]. The cell suspensions were incubated at 37 °C with 5% CO_2_ for 4 h to allow stimulation. Surface staining was performed first, which was followed by permeabilization using P/W for 15 min at 4 °C. The subsequent intracellular staining followed the same protocol as the previously described surface staining process, with all antibodies incubating for 25 min at 4 °C. After each antibody incubation, the cells were washed with P/W. After the final P/W wash, cells were rinsed with SB and then resuspended in 100 μL SB. Isotype controls were stained using isotype antibodies, and an unstained control was included following the same protocol. Flow cytometry was performed, acquiring a minimum of 20,000 events. Data analysis was conducted using FlowJo version 10 (Tree Star, Ashland, OR, USA).

### 2.6. Statistical Analysis

Statistical analyses were performed with Prism 8 (GraphPad Software, Inc., La Jolla, CA, USA); specific details thereof are provided in the figure legends. Overall, all data have passed the Anderson–Darling normality test. All data were analyzed using one-way ANOVA with Tukey’s Multiple Comparisons Test. Asterisks indicate statistical significance. * *p* < 0.05; ** *p* < 0.01; *** *p* < 0.001; **** *p* < 0.0001.

## 3. Results

### 3.1. Similar Levels of TCRγδ^+^ and TCRαβ^+^ T Cells in Bovine T-IELs

**Figure 1:**
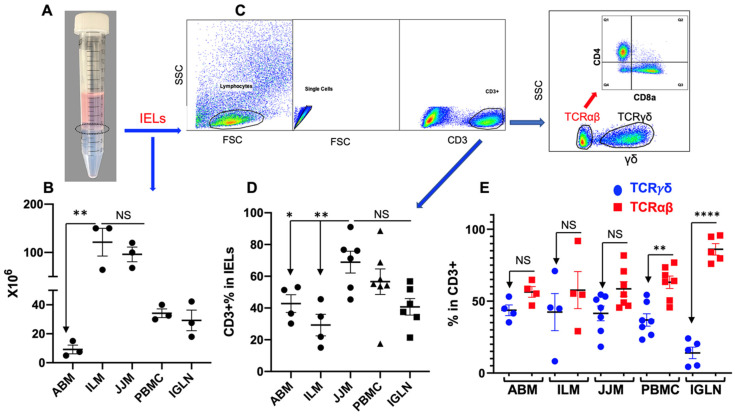
Similar levels of TCRγδ^+^ and TCRαβ^+^ T cells in bovine T-IELs. ABM, JJM, ILM, blood, and inguinal lymph node (IGLN) were harvested from finished steers as described in our previous report [32]. ABM: abomasal mucosa. ILM: ileum mucosa. JJM: jejunum mucosa. PBMC: peripheral blood mononuclear cells. A-B: T-IELs collected from the interface (**A**), and comparison of their yield per unit (**B**): ABM (~100 g), ILM and JJM (both 10 cm in length), PBMC (10 mL of blood), and IGLN (~2 g). (**C**) Gating strategies: CD3^+^ cells were gated based on single lymphocytes, which were further separated into TCRγδ^+^ and TCRγδ^−^, representing TCRγδ^+^ and TCRαβ^+^ T cells. The TCRαβ^+^ (TCRγδ^−^) population was further analyzed for CD4^+^ and CD8α^+^. D-E. Comparison of T cells (CD3^+^) in isolated T-IELs (**D**), and TCRγδ^+^ and TCRαβ^+^ T cells in T-IELs, PBMC, and IGLN (**E**). The data were presented as the mean of the individual cattle plus the standard error. This data presentation will be the same in the rest of the figures. All data passed the Anderson–Darling normality test and were analyzed using one-way ANOVA with Tukey’s Multiple Comparisons. Asterisks indicate statistical significance. * *p* < 0.05; ** *p* < 0.01; **** *p* < 0.0001. “NS” indicates not significant. This statistical analysis and these indications will be applied throughout the rest of this manuscript.

Tissues from the blood and inguinal lymph nodes were collected from finished steers as in our previous report [32]. The protocol for T-IEL isolation was based on previous research but with some modifications [15,21,32]. After processing the samples, T-IELs were retrieved from the interface (Figure 1A), washed, and prepared for subsequent procedures. The yield of T-IELs was abundant, about 100 × 10^6^ in the ileum mucosa (ILM) and the jejunum mucosa (JJM) from a 10 cm segment of each, which dropped by about 90% in abomasum mucosa (ABM) (~100 g) (Figure 1B). The low frequency of T-IELs in ABM has been observed previously [21]. T cell fraction, as indicated by CD3^+^ staining, was about 70% in JJM but was significantly lower in both ABM and ILM (Figure 1D), which suggests that the T cell fraction may vary across different segments of the GI tract. Since more than 90% of T-IELs express either TCRγδ or TCRαβ, we used the exclusion of TCRγδ as the marker for TCRαβ in T-IELs (as there is no TCRαβ antibody available for cattle) (Figure 1C). The frequency of TCRαβ^+^ T-IELs was not different from that of TCRγδ^+^ T-IELs. However, TCRαβ^+^ T cells were in a higher proportion compared to TCRγδ^+^ T cells in PBMC and IGLN (Figure 1E). This even distribution of TCRαβ^+^ and TCRγδ^+^ T-IELs in cattle is different from that in humans (>85% TCRαβ^+^ T-IELs), but comparable to mice [79].

### 3.2. TCRγδ^+^ T-IELs Are Dominantly CD8-Negative

**Figure 2:**
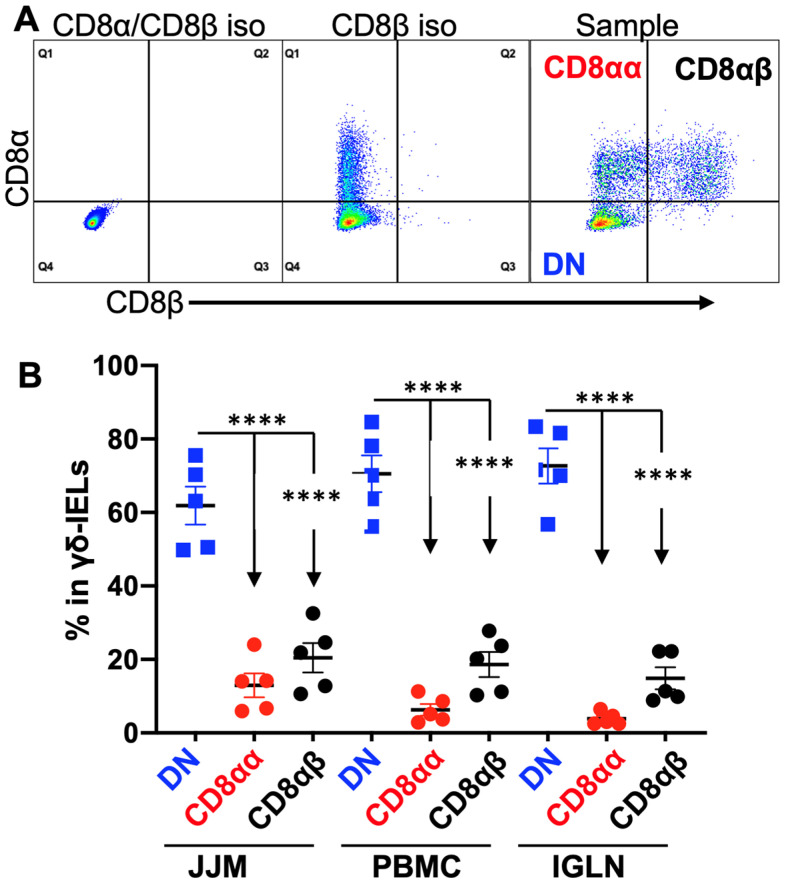
TCRγδ^+^ T cells are dominantly CD8-negative in T-IELs. (**A**) Gating strategies for CD8α^+^ and CD8β^+^ expression in TCRγδ^+^ T cells are based on CD3^+^ as indicated in Figure 1C. DN: double negative for CD8α and CD8β. CD8α^+^ and CD8β^−^ were defined as CD8αα^+^, according to previous reports [137,138]. Iso: isotype antibody control. (**B**) Comparison of CD8α^+^ and CD8β^+^ expression in TCRγδ^+^ T cells from different tissues. Each population (CD8αα and CD8αβ) was indicated in the dot plot for the “Sample” in panel A. **** *p* < 0.0001.

TCRγδ^+^ T-IELs make up a small population of T-IELs in humans (<15%), but a major proportion in mice (40–60%) [79]. In adult cattle, TCRγδ^+^ T-IELs were approximately 40% of T-IELs (CD3^+^) (Figure 1E), comparable to those in mice. Nonetheless, most murine TCRγδ^+^ T-IELs are CD8αα^+^ (>75%); bovine TCRγδ^+^ T-IELs were predominantly (>60%) CD8-negative (Figure 2A,B), and a small percentage of TCRγδ^+^CD8^+^ T-IELs expressed both CD8 subunits (CD8αβ^+^) or only the CD8α^+^ subunit (CD8αα^+^) (Figure 2B) as defined using established methods [137,138]. TCRγδ^+^ T cells are abundant in the blood of young calves, and decrease with age [120]. In addition, CD8-negative TCRγδ^+^ T cells from the blood have been extensively studied, and are recognized for both their immune stimulatory and regulatory functions [120,139,140,141,142,143,144]. We plan to investigate whether the composition and function of TCRγδ^+^ T-IELs changes with age in a manner similar to that in the blood.

### 3.3. TCRαβ^+^CD4^+^CD8αβ^+^ T Cells Are Substantial in T-IELs but Not in the Blood and Lymph Nodes

**Figure 3:**
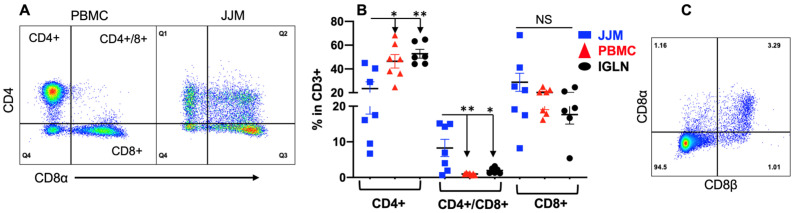
TCRαβ^+^CD4^+^CD8αβ^+^ T cells are substantial in the T-IEL population but not in the blood and lymph nodes. TCRαβ^+^ were based on CD3^+^ and TCRγδ^−^ as indicated in Figure 1C. (**A**) Representative dot plots and gating strategies for CD4^+^ and CD8^+^ analysis based on TCRαβ^+^. CD8^+^ was indicated via CD8α staining. (**B**) Comparison of subpopulations based on TCRαβ^+^CD4^+^ or TCRαβ^+^CD8^+^ in total CD3^+^ lymphocytes. (**C**) Representative dot plots of CD8α and CD8β expression in CD4^+^/CD8^+^ TCRαβ^+^ T-IELs in (**B**). * *p* < 0.05; ** *p* < 0.01. “NS” indicates not significant.

TCRαβ^+^ T-IELs constitute a major portion of T-IELs in humans (>80%) and a significant portion in mice (>30%) [79]. Indeed, bovine TCRαβ^+^ T-IELs expressing either CD4 and/or CD8α made up more than 50% of the total T-IELs (CD3^+^) across all the tissues (Figure 3A,B). Furthermore, the percentages of CD4^+^ and CD8α^+^, which are the single positive T-IELs in JJM, were nearly equal (~20% of CD3^+^), and collectively reached the number of TCRγδ^+^ cells in T-IELs (CD3^+^) (~40%) (Figure 1E and Figure 3B). The frequency of CD4^+^ in T-IELs was lower in JJM than in PBMC and IGLN (Figure 3B). Notably, there was a subpopulation of CD4^+^CD8α^+^ (double positive) TCRαβ^+^ T-IELs in the mucosa, which was almost absent in the blood and IGLN (Figure 3B). These TCRαβ^+^CD4^+^CD8α^+^ T-IELs were predominantly CD8αβ^+^ (Figure 3C), so they are different from the TCRαβ^+^CD4^+^CD8αα^+^ T-IELs in mice and the TCRαβ^+^CD4^+^ or TCRαβ^+^CD8αβ^+^ T-IELs in humans [145]. The TCRαβ^+^CD4^+^CD8αβ^+^ T-IELs may have combined function of TCRαβ^+^CD4^+^ and TCRαβ^+^CD8αβ^+^ T-IELs, potentially playing a crucial role in controlling GI extracellular pathogens and in exerting cytotoxicity to infected epithelial cells, which will be further investigated in the future [61,79,108,146,147]. Furthermore, the CD8α^+^ single positive TCRαβ^+^ T-IELs were also CD8β^+^ across all bovine tissues (Supplementary Figure S1), suggesting an absence of TCRαβ^+^CD8αα^+^ T-IELs, a profile different from that seen in humans and mice.

### 3.4. CD69 Is Highly Expressed in T-IELs, While CD62L Is Expressed at a Lower Level Compared to Their Counterparts in the Blood or Lymph Nodes

**Figure 4:**
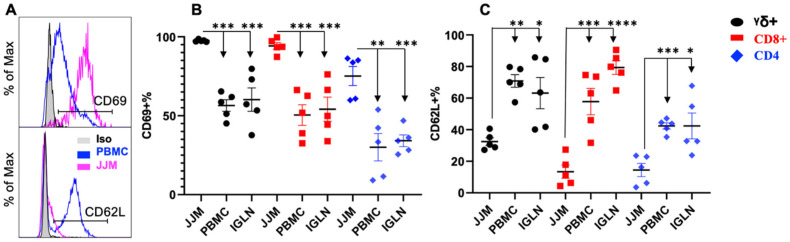
CD69 and CD62L are differentially expressed in T-IELs compared to T cells in PBMC and lymph nodes. TCRαβ^+^CD8^+^ and TCRαβ^+^CD4^+^ T cells were based on CD3^+^ and TCRγδ^−^ as indicated in Figure 1C. (**A**) Gating strategies for CD69 and CD62L expression. B-C: comparison of CD69 (**B**) or CD62L (**C**) expression on subpopulation TCRγδ^+^, TCRαβ^+^CD8^+^, and TCRαβ^+^CD4^+^ T cells in T-IELs, PBMC, and IGLN. * *p* < 0.05; ** *p* < 0.01; *** *p* < 0.001; **** *p* < 0.0001.

CD69 is a marker for T-IEL activation [148] and is also associated with tissue residency [149,150]. Conversely, CD62L, also known as L-selectin, is linked with homing into secondary lymphoid tissues, especially lymph nodes [151]. T-IELs are believed to be in an activated-yet-resting state, allowing them to respond to innate signals beyond those from the TCR [1]. Indeed, CD69 expression was greater in TCRγδ^+^, TCRαβ^+^CD8α^+^, and TCRαβ^+^CD4^+^ T-IELs compared to those in PBMC and IGLN (Figure 4A,B), indicating their tissue residency and activation status. TCRαβ^+^CD4^+^ T-IELs exhibited significantly less CD69 expression than the TCRγδ^+^ and TCRαβ^+^CD8α^+^ T-IELs (Figure 4B). Consistently, T-IELs from JJM had the lowest expression of CD62L compared to T cells from PBMCs and IGLN, indicating distinct homing patterns. Among T-IELs, TCRαβ^+^CD8^+^ and TCRαβ^+^CD4^+^ cells had lower levels of CD62L than TCRγδ^+^ (Figure 4C). The subunit of the IL-2 receptor, CD25, is a characteristic activation marker for conventional T cells [152,153]. CD25 is enhanced in bovine CD4^+^ T cells in response to TCR stimulation in vitro [32], and is expressed at a low level on CD4^+^ and TCRγδ^+^ T cells from the blood, but is virtually absent in CD8^+^ T cells [121]. To determine whether the expression of CD25 is associated with the activation status of T-IELs, the CD25 expression of various subsets of T-IELs from JJM was compared to that of their blood and IGLN counterparts. Consistent with our previous report [121], among T-IELs, TCRαβ^+^CD4^+^ and TCRγδ^+^ had modest but significantly higher levels of CD25 than TCRαβ^+^CD8^+^ (Supplementary Figure S2), following a similar trend in the blood and IGLN. Moreover, the expression of CD25 in T-IELs was similar to that in blood and IGLN T cells (Supplementary Figure S2), indicating that CD25 expression may not be used as an indicator of activation status in T-IELs as it is in conventional T cells. This suggests that IL-2 signaling may not be critical for the maintenance of these half-activated T-IELs, but does not exclude its importance in their activation such as after TCR stimulation.

### 3.5. T-IELs Are Able to Produce Cytokines

**Figure 5:**
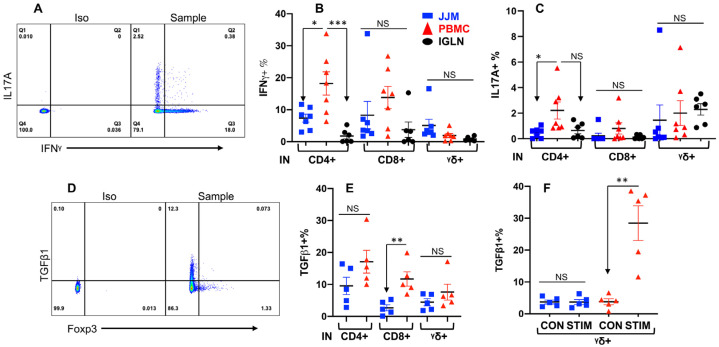
T-IELs are able to produce cytokines. Single-cell suspensions from different tissues were incubated for 4 h with an activation cocktail [121] before staining for IFNγ (**B**) and IL17A (**C**) or TGFβ1 (**G**). (**A**–**C**) Gating (**A**) and comparison of IFNγ (**B**) and IL17A (**C**) production in TCRαβ^+^ T cells (CD4^+^ or CD8^+^) and TCRγδ^+^ T cells as indicated in Figure 1C. Iso: Isotype antibody control. (**D**) Gating strategy for TGFβ1 expression based on TCRαβ^+^CD4^+^ T-IELs. (**E**) Direct staining for TGFβ1 in fresh samples without further stimulation. (**F**) Comparison of TGFβ1 expression in samples after stimulation as in (**A**–**C**). CON: no stimulation control in culture. STIM: stimulated. Colors indicate tissues in (**B**,**C**,**E**,**F**). * *p* < 0.05; ** *p* < 0.01; *** *p* < 0.001. “NS” indicates not significant.

Cytokines play an important role in regulating immune responses. To test whether T-IELs could be induced to produce cytokines, these cells were stimulated with an activation cocktail as described in our previous reports [32,121], using PBMC and IGLN as controls. Indeed, all T-IEL subsets produced IFNγ and a trace of IL17A, with TCRαβ^+^CD4^+^ T-IELs producing less than their PBMC counterparts (Figure 5A–C). IFNγ-producing T-IELs were rarely stained positive for IL17A (Figure 5A), suggesting functionally distinct subpopulations, with some producing IFNγ and others producing IL17A. The production of IFNγ and IL17A was undetectable without induction by the cocktail (data not shown). Since only activated or memory T cells are able to produce IFNγ and IL17A in response to short-time stimulation, these data support that bovine T-IELs are semi-activated, similar to those in humans and mice [154,155]. Immune regulatory cytokines such as TGFβ1 play a significant role in maintaining the homeostasis of the epithelial barrier [1]. Usually, TGFβ1 has to be induced by the cocktail stimulation before detection of T cells in humans, mice, and cattle [139,156,157]. To our surprise, TGFβ1 was detected in all subpopulations in fresh samples (without stimulation) (Figure 5E), suggesting the constitutive production of TGFβ1 in T-IELs and T cells in other tissues. Further stimulation of T-IELs induced enhanced TGFβ1 production in the TCRγδ^+^ T cells within PBMCs but induced no response in the TCRαβ^+^ T cells within PBMCs or in any T-IEL subtypes (Figure 5F and Supplementary Figure S3). TGFβ1-producing TCRαβ^+^CD4^+^ T-IELs were rarely stained positive for Foxp3, a Treg marker (Figure 5D), implying that TGFβ1-producing TCRαβ^+^CD4^+^ T-IELs are not Tregs. These data confirm that different subsets of bovine T-IELs differ in their functions from their counterparts in the blood and lymphoid tissues, as demonstrated by cytokine production differences.

## 4. Discussion

Bovine T-IELs, while demonstrating certain similarities, evidently differ from their counterparts in humans and mice. These variations are particularly noticeable in the distribution of the TCRγδ^+^ and TCRαβ^+^ T-IEL subsets. Uniquely, subsets such as TCRαβ^+^CD4^+^CD8αβ^+^ and TCRγδ^+^CD8^−^ are present in bovine T-IELs, while the TCRαβ^+^CD8αα^+^ T-IELs are notably absent. Despite these distinct features, bovine T-IELs continue to demonstrate key immunological functions analogous to those in humans and mice, specifically in their pre-activated states and with the production of immune regulatory as well as inflammatory cytokines, suggesting similar functions in maintaining gut homeostasis and pathogen control. This is the first time that T-IELs in the bovine gut have been characterized, furthering our understanding of bovine mucosal immunity and offering insights into the development of innovative drugs and vaccines against mucosal diseases.

Variations in the number and proportions of T-IELs might stem from the diverse GI microenvironments and age-related differences across species [116]. While direct evidence is still lacking, there are indications that dietary compounds/residues affect T-IEL numbers [43,46,48,50,58,158,159,160,161,162,163,164,165,166,167,168,169,170,171]. For example, the aryl hydrocarbon receptor in T-IELs is vital for maintaining their population, and its deficiency reduces T-IEL numbers [172]. Furthermore, the proportions of different T-IEL subtypes within intestinal segments are significantly influenced by age. For instance, younger individuals typically display higher proportions of natural T-IELs, but this balance shifts towards induced T-IELs with age in humans and mice [1,79,98]. This age-related trend is also observed in cattle, which is demonstrated by the dominance of TCRγδ^+^ T-IELs in the ileum immediately post-birth and during early life and the presence of higher proportions of CD4^+^ and CD8^+^ T-IELs in weaned calves compared to those in suckling calves [20,173,174]. Our studies in cattle have shown comparable proportions of TCRγδ^+^ and TCRαβ^+^ T-IELs. As bovine peripheral TCRγδ^+^ T cells inhibit the proliferation of TCRαβ^+^ T cells by producing IL-10 and TGFβ1 and the depletion of TCRγδ^+^ T cells results in enhanced proliferation of antigen-specific TCRαβ^+^ T cells in cattle infected with *Mycobacterium bovis* [139,140,141,142,143], we postulate that an initial dominance of natural TCRγδ^+^ T-IEL in young calves could assist in tolerating non-harmful antigens. In contrast, adult cattle may host a larger proportion of induced T-IELs in the gut due to increased antigen exposure over time. Additionally, induced T-IELs constitute a large proportion of the total T-IELs in humans, while mice maintain a significant proportion of natural T-IELs [79]. It could be hypothesized that species with longer lifespans might host a larger proportion of antigen-stimulated induced T-IELs. In conclusion, species-specific GI microenvironments and age-related shifts should significantly impact the proportions and functions of T-IEL subsets.

The unique TCRγδ^+^CD8^−^ subset in bovines potentially plays a crucial role in pathogen defense and immune homeostasis. Bovine TCRγδ^+^ T-IELs are predominantly CD8-negative, with only a small fraction expressing either CD8αβ and/or CD8αα. In contrast, mouse TCRγδ^+^ T-IELs mainly express CD8αα [42,79,116,175]. CD8αβ^+^ is associated with cytotoxicity, while CD8αα is linked to immune suppression and cell survival [98,176,177]. For example, human CD8αβ^+^ T-IELs exhibit cytotoxic responses and can enhance T cell sensitivity to a cognate antigen by 100-fold compared to their CD8αα-expressing counterparts [98,176]. Conversely, CD8αα represses TCR stimulation by avoiding integration into the TCR complex lipid raft and can prevent the exhaustion of chronically activated CD8^+^ T cells or excessive cytolytic responses, thus serving as an immunosuppressive and survival-aiding molecule in mice [98,176,177]. This evidence suggests that the minor population of bovine TCRγδ^+^ T-IELs expressing CD8αβ likely plays a dominant role in defending against pathogens, whereas those expressing CD8αα might promote immune homeostasis. Moreover, the TCRγδ^+^CD8^−^ population is also observed in the blood, peripheral lymph nodes, skin, and spleen marginal zones in cattle [178]. Despite the TCRγδ^+^CD8^−^ T cell subset being known to display inflammatory functions in response to various infectious agents such as *Anaplasma marginale* and *Mycobacterium bovis* in the bovine periphery [120,144], the same population is also a major regulatory T cell subset [139,140,141,142,143]. This resembles our findings, where the TCRγδ^+^ subpopulation in T-IELs and in blood expressed low levels of TGFβ1. However, the function of the TCRγδ^+^ T-IELs may differ from their counterparts in the blood, as further stimulation enhanced TGFβ1 production only in those from the blood, not those from T-IELs. We predict that TCRγδ^+^ T-IELs play a crucial role in maintaining intestinal immune tolerance and also mediate certain immune responses. Additionally, the absence of the TCRαβ^+^CD8αα^+^ subset in cattle suggests a potential divergence in their immunological profile compared to humans and mice, implying the possibility of compensation through alternative subsets. Under normal conditions, TCRαβ^+^CD8αα^+^ T-IELs constitute a significant portion of T-IELs in mice and perform immunomodulatory functions, supported by a wide array of NK receptor expressions, including NKG2A/CD94 and the Ly49 members [61,79]. In contrast, the TCRαβ^+^CD8αβ^+^ T-IELs in mice are geared towards immune responses and cytolytic functions, demonstrated by their substantial capacity to produce cytokines such as IFNγ and express high levels of granzyme B [61,79]. Interestingly, humans have a minimal proportion of TCRαβ^+^CD8αα^+^ T-IELs, while the TCRαβ^+^CD8αβ^+^ subset exhibits the dual capacity for immunomodulation and cytotoxicity by expressing NK receptors and displaying the potential to produce IFNγ and granzyme B [79]. We might also anticipate similar dual functionalities of TCRαβ^+^CD8αβ^+^ T-IELs in cattle. Given these findings, it is worth considering that other bovine T-IEL subsets such as TCRγδ^+^CD8^−^, TCRγδ^+^CD8αα^+^, and/or TCRαβ^+^CD4^+^ T-IELs might compensate for the immune regulatory role of the TCRαβ^+^CD8αα^+^ T-IELs found in mice. However, further research is required to generate confirmatory results.

The significant presence of distinct TCRαβ^+^CD4^+^CD8αβ^+^ T-IELs in cattle may possess the combined characteristics of both CD4^+^ and CD8αβ^+^ T-IELs. In mice, various GI microenvironment factors such as food components like retinoic acid and gluten, as well as cytokines like TGFβ1 and IFNγ, can downregulate the CD4 lineage transcription factor ThPOK and upregulate the CD8 lineage transcription factor Runx3, which leads to the expression of CD8αα in CD4^+^ T-IELs [1,179,180,181,182]. In this context, both TCRαβ^+^CD4^+^CD8αα^+^ and TCRαβ^+^CD4^+^ T-IELs contribute to immune tolerance [108,109,146,147,179,183]. Although CD8α induction in TCRαβ^+^CD4^+^ T-IELs has been well established [181,184,185], it is atypical for mature T cells to switch the CD4 and CD8αβ coreceptors once they have differentiated into the CD4 or CD8αβ lineage post positive selection in the thymus. For instance, a CD4^+^ T cell will generally not express CD8αβ, and vice versa [186]. At present, we speculate that this unique TCRαβ^+^CD4^+^CD8αβ^+^ T-IEL subset in cattle might possess both the immunoregulatory functions of CD4^+^ and the cytotoxicity of CD8αβ^+^ T-IELs. Nonetheless, this concept warrants further studies.

Bovine T-IELs exhibit an activated-yet-resting state in the gut, similar to their human and mouse counterparts, but with potential differences. CD62L is essential for T cell adhesion, facilitating lymphocyte homing to secondary lymphatic organs. In contrast, CD69 serves a dual role: it counters the sphingosine-1-phosphate receptor, which typically signals to T cells to move into the bloodstream, and acts as a T cell activation marker [149,150,187,188,189]. Bovine T-IELs express low levels of CD62L and high levels of CD69, a pattern also observed in humans and mice [61,190]. Moreover, the TCRγδ^+^ and TCRαβ^+^CD8αα^+^ T-IELs express substantially lower levels of CD62L compared to their PBMC and IGLN counterparts (Figure 4C). This variation suggests that T-IELs might utilize distinct adhesion and migration molecules, consistent with their roles in retention and surveillance within epithelial barriers [1]. Furthermore, minuscule populations of T-IELs in humans, mice, and cattle produce IFNγ and IL17A under normal conditions. Upon stimulation, these T-IEL populations increase their production of both cytokines [185,191,192,193]. Notably, all T-IEL subsets in humans and mice include an IFNγ/IL17A co-producing population [185,191,192,194]. In cattle, however, the populations that produce IFNγ and IL17A are distinct and do not overlap, potentially representing functionally different subpopulations. In summary, while bovine T-IELs display residency and activation markers comparable to those in human and mouse T-IELs, cattle exhibit unique T-IEL populations for IFNγ and IL17A production, suggesting potential functional variances.

Bovine T-IELs, similar to those in humans and mice, secrete low amounts of the immune regulatory cytokine TGFβ1, which helps maintain immune tolerance under normal conditions [24,195,196,197,198,199]. TGFβ1 exerts several effects, such as downregulating IFNγ production, enhancing CD103 expression in Tregs, strengthening epithelial tight junctions, promoting the differentiation of goblet cells, paneth cells, and tuft cells, and stimulating fibrosis to reduce inflammation [199,200,201,202]. These effects promote immune tolerance and epithelial barrier protection in humans and mice. In our study, similar to humans and mice, all subsets of bovine T-IELs, particularly the TCRγδ^+^ subpopulation, produced TGFβ1 under normal conditions, suggesting similar functions in cattle. Furthermore, stimulation enhanced TGFβ1 production only in TCRγδ^+^ T cells in the PBMC in cattle, indicating the regulatory role of this subset in systemic circulation during immune reactions as previously found [139,140,141,142,143]. In summary, our findings suggest that bovine T-IELs possess the ability to maintain homeostasis by producing the immune regulatory cytokine TGFβ1.

Our research shows that bovine T-IELs differ from those in humans and mice, making it of paramount importance to understand their roles in cattle. Although we characterized the bovine T-IELs by analyzing their surface and intracellular molecules, we have not yet tested their functions such as how they respond to different stimuli, which is currently an ongoing project in the lab.

## 5. Conclusions

Our study reveals the unique composition and potential roles of T-IELs for the first time in cattle, highlighting functional conservation across species as well as specialized adaptations. These findings open avenues for further investigations into bovine T-IELs and their role in developing strategies against gastrointestinal disorders including both acute and chronic infections in cattle. Our next steps involve functional assays and molecular analyses to further understand the mechanisms by which bovine T-IELs contribute to maintaining homeostasis and immune responses in cattle.

## Data Availability

Data are contained within the article and supplementary material.

## Supplementary Materials

The following supporting information can be downloaded at https://www.mdpi.com/article/10.3390/pathogens12091173/s1, Figure S1: TCRαβ^+^CD8^+^ T-IELs express both CD8α and CD8β; Figure S2: CD25 is higher in TCRαβ^+^CD4^+^ T-IELs than in TCRαβ^+^CD8^+^ T-IELs; Figure S3: Activation does not affect TGFβ1 expression in TCRαβ^+^CD4^+^ or TCRαβ^+^CD8^+^ T-IELs; Table S1: Primary antibodies; Table S2: Secondary antibodies and isotype controls.
