# Supplementary material for: Characterization of Bovine Intraepithelial T Lymphocytes in the Gut
Source: Pathogens. 2023 Sep 19;12(9):1173. doi: 10.3390/pathogens12091173 (PMC10535955; doi:10.3390/pathogens12091173)
Supplement: Supplementary file 1 [file pathogens-12-01173-s001.zip › pathogens-2598506-supplementary.pdf]

Characterization of bovine intraepithelial T lymphocyte in the gut

Akanksha Hada[1], Lei Li[1], Anmol Kandel[1], Younggeon Jin[1], and Zhengguo Xiao[1]

[1]Department of Animal and Avian Sciences, University of Maryland, College Park, Maryland 20742, USA

Corresponding Author: Zhengguo Xiao, Ph.D.
Department of Animal and Avian Sciences
University of Maryland
College Park, MD 20742 USA
Phone: 301-405-6258
FAX: 301-405-7980
Email: xiao0028@umd.edu

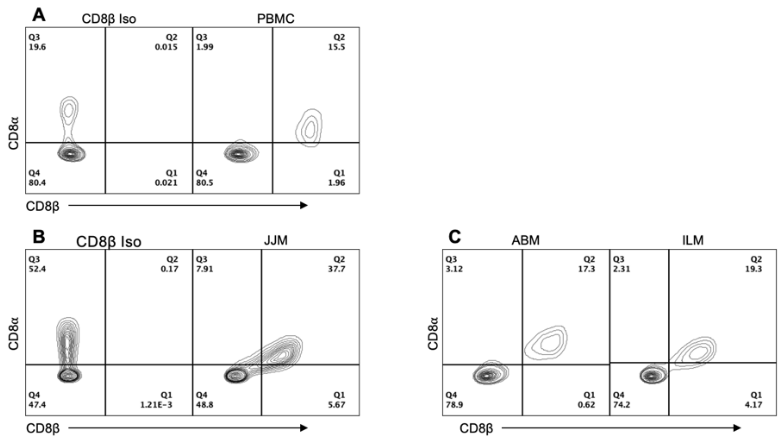

Supplementary Figure S1: TCRαβ$^+$CD8$^+$ T-IELs express both CD8α and CD8β. Isolated IELs were stained with anti-bovine CD3, TCRγδ, CD8α, and CD8β. The counterplot was based on CD3$^+$TCRγδ$^-$ cells, using the 5% possibility in Flowjo setting. A: Expression of CD8α and CD8β in TCRαβ$^+$ cells from PBMCs. Isotype control was anti-CD8β only since anti-CD8α is the most commonly used antibody. B: Expression of CD8α and CD8β in TCRαβ$^+$ T-IELs isolated from JJM. C: Expression of CD8α and CD8β in TCRαβ$^+$ T-IELs isolated from ABM and ILM. These are representatives from three experiments.

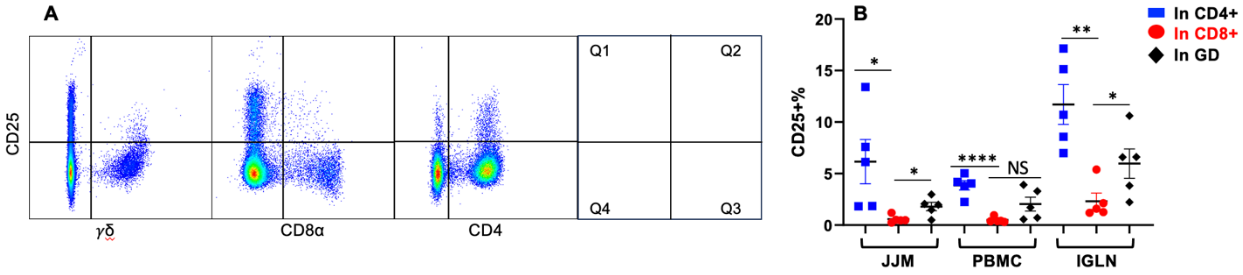

Supplementary Figure S2: CD25 is higher in TCRαβ$^+$CD4$^+$ T-IELs than in TCRαβ$^+$CD8$^+$ T-IELs. Isolated IELs were stained with anti-bovine CD3, TCRγδ, CD4, CD8α, and CD8β. Gating for TCRγδ$^+$ T-IELs or TCRγδ$^+$ T cells in PBMCs were based on CD3$^+$TCRγδ$^+$. Gating for TCRαβ$^+$CD4$^+$ or TCRαβ$^+$CD8$^+$ T-IELs or TCRαβ$^+$ T cells in PBMCs were based on CD3$^+$TCRγδ$^-$. A. Gating strategy for CD25 expression. The percentage of CD25$^+$ (CD25$^+$%) was calculated based on Q2/(Q2+Q3) x 100%. B. Comparison of CD25$^+$% on TCRαβ$^+$CD4$^+$CD8$^+$ T-IELs and TCRγδ$^+$ T-IELs (GD) in different tissues.

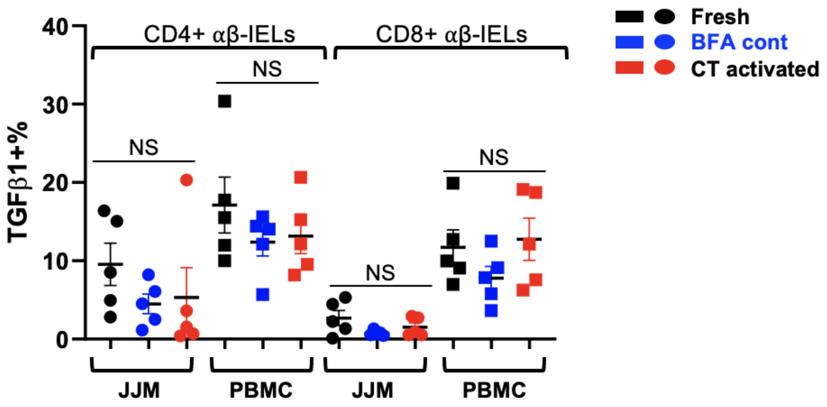

Supplementary Figure S3: Activation does not affect TGFβ1 expression in TCRαβ$^+$CD4$^+$ or TCRαβ$^+$CD8$^+$ T-IELs. T-IELs were gated and analyzed in the same manner as in Fig. 5F-G. BFA: Brefeldin A.

Supplementary Table S1. Primary antibodies.

Specificity	Clone	Purified Primary Antibody Isotype	Source
bCD3	MM1A	IgG1	WSUMAC
bTCRδ	GB21A	IgG2b	WSUMAC
bCD8b	BAT82A	IgG1	WSUMAC
bCD69	KTSN7A	IgG1	WSUMAC
bCD25	LCTB2A	IgG3	WSUMAC
bIL4	CC303	IgG2a	Bio-Rad

Specificity	Clone	Conjugated Antibody Isotype	Source
bCD4	CC8	IgG2a-FITC	Bio-Rad
bCD8	CC63	IgG2a-RPE	Bio-Rad
bCD8	CC63	IgG2a-FITC	Bio-Rad
bCD62L	CC32	IgG1-FITC	WSUMAC
bIFNγ	CC302	IgG1-PE	Bio-Rad
hIL-17A	eBio64DEC17	IgG1-APC	Invitrogen
hTGF-β1	TW4-2F8	IgG1-PE	BD Biosciences
rFoxP3	FJK-16s	IgG2a-PE	Invitrogen

* b, Bovine. h, Human. r, Rat. WSUMAC, Washington State University Monoclonal Antibody Center. Bio-Rad, Bio-Rad Laboratories, Inc. Hercules, CA, USA.

Supplementary Table S2. Secondary antibodies and isotype controls.

Specificity	Secondary antibodies	Source
IgG1	Anti-mouse IgG1-Biotin	BioLegend

IgG1	Anti-mouse IgG1-BV421	BioLegend
IgG2a	Anti-mouse IgG2a-APC	BioLegend
IgG2a	Anti-mouse IgG2a-BV421	BioLegend
IgG2a	Anti-mouse IgG2b-Biotin	BioLegend
IgG2a	Anti-mouse IgG2a-PerCP	BioLegend
IgG2b	Anti-mouse IgG2b-APC	BioLegend
IgG3	Anti-mouse IgG3-Biotin	BioLegend
IgG3	Anti-mouse IgG3-BV421	BioLegend
Biotin	Streptavidin Per-CP	BioLegend

**Isotype control antibodies**	**Source**
Mouse IgG1	BioLegend
Mouse IgG2a	BioLegend
Mouse IgG2b	BioLegend
Mouse IgG3	BioLegend
Mouse IgG1-APC	BioLegend
Mouse IgG1-PE	BioLegend
rat-IgG2a-APC	BioLegend
rat-IgG2a-PE	BioLegend
